# Location of the Cut Flexor Tendons in the Palm Using Surface Anatomy: A Simple Guide for Minimal Incision Surgery

**DOI:** 10.29252/wjps.9.3.321

**Published:** 2020-09

**Authors:** Afshin Fathi

**Affiliations:** Department of Plastic and Reconstructive Surgery, Tehran University of Medical Sciences, Tehran, Iran

**Keywords:** Flexor tendon, Tendon injury, Surface anatomy, Palm, Surgery

## Abstract

**BACKGROUND:**

Hand Zone 2 flexor injuries are among the most important tendon lesions and its prognosis is poorer than the other hand zones. Limited incisions prevent multiple skin flaps. The present study aimed to determine the location of palmar flexor tendons using surface anatomical markers to provide a simple and easy guide for hand surgeons assisting them in minimal incisions.

**METHODS:**

Patients with hand injuries in Zone 2 were taken to the operating room for surgery. The skin and subcutaneous tissue over the flexor tendons sheath were incised. After finding the flexor tendons in the palm, their exact position was located with a blue needle (23G) over the palm crease and marked relative to the finger borders.

**RESULTS:**

Thirty-eight patients with the mean age of 27±3.6 years were operated. Totally, 57 fingers and 38 palms were dissected. The flexor tendons were located under the proximal and distal palm creases between two parallel lines extended from finger borders and mid-axial axis of the fingers. The average distance from finger print to distal palmar crease was 25, 32 and 24 mm for little, ring and long finger, respectively and 32 mm from index finger print to mid palmar crease.

**CONCLUSION:**

The precise position of flexor tendons can be easily determined in the palm according to surface anatomical markers and plan for limited incisions.

## INTRODUCTION

Hand Zone 2 flexor injuries are among the most important tendon lesions. Exploration of this zone can lead to further damage to its components, such as pulleys, and increase the risk of postoperative complications. Adhesion at the repair site is the most common complication.^1^^,^^2^ On the other hand, the cut tendons in Zone 2 lack the proximal end, which is retracted towards the palm, requiring further steps to pull it back to the cut site. In this regard, Zone 2 is referred to as the “no man’s land”.^3^

Some authors have proposed different methods to access the injury site. In 1944, Bunnell introduced the midaxial incision of flexor tendons;^4^ the midlateral incision by Varden,^5^ and in 1967, Bruner explained the zigzag volar-digital incision.^6^ Despite the simplicity and appropriate exposure of the Bruner method, the risk of iatrogenic damages is high due to the large dissection required, when the palm undergoes exploration. In general, the prognosis of Zone 2 injuries is poorer than the other hand zones.^3^

Limited incisions prevent multiple skin flaps. However, these methods require knowledge of finger and hand surface markers to determine the exact location and anatomy of the tendons. The location of finger flexor tendons and pulleys was already described.^7^ The present study aimed to determine the location of palmar flexor tendons using surface anatomical markers to provide a simple and easy guide for hand surgeons assisting them in minimal incisions.

## MATERIALS AND METHODS

Patients with hand injuries in Zone 2 who referred to our emergency department from December 10, 2017 to March 3, 2019 were enrolled in this study. Informed consent was received and the rights of the patients were protected. After transferring them to operating room, the patients were prepared through nerve blocking and sedation or general anesthesia and, using a tourniquet if necessary. Flexor tendons and pulleys were explored through the Bruner method with zigzag incision up to the distal or middle palm to find the proximal ends of tendons.

After finding the flexor tendons in the palm, their exact position was located with a blue needle (23G) over the palm crease and marked relative to the finger borders. Then, the proximal end of the cut tendon was attached to a small tube (G8) or a straightened silk needle 2-0, which was already passed from the cut site beneath the A_1_ and A_2_ pulleys up to the palm, and was slowly pulled back distally beneath the pulleys with a proper maneuver. After repairing the tendon using the Kessler method, the skin flaps were sutured in the correct positions. Then, in order to identify the exact location of flexor tendons in the palm, the designated areas on the palm and its creases, as well as the distance between the tendons relative to the radial, ulnar, and midiaxial edges of each finger were measured.

## RESULTS

In this study, 57 fingers and 38 palms underwent an operation in 38 patients, 29 men (76%) and 9 women (24%) with a mean age of 27±3.6 years. The causes of injuries were cutting with a knife or glass in the kitchen, sharp object at the workshop, motor vehicle accident and during the conflict. Thirty-four (89.47%) patients were male (*p*<0.0001). The mean footprint of four fingers and the mean distance between the proximal finger creases to the palm creases were presented in Table 1. 

**Table 1:** Surface markers of fingers and palm

**Surface marker**	**Finger**			
	**Index (Mean±SD** ^*^ **)**	**Long**	**Ring**	**Small**
Finger foot print	23.44±2.02	25.60±1.65	23.98±1.97	22.57±1.34
MAD^**^	26.14±1.63	26.63±3.05	25.57±1.34	30.02±1.57
UBD^***^	23.45±2.05	30.24±1.65	29.98±1.44	31.75±1.14
RBD^****^	29.68±1.66	21.74±1.32	12.01±3.26	30.17±1.44

*SD: Standard Deviation, **MAD: Mid-axial distance, the distance between mid-point of finger print to distal palmar crease; ***UBD: Ulnar border distance, the distance between ulnar border of finger print to palmar crease; ****RBD: Radial border distance. For Index finger the distances are from mid-palmar crease

**Fig. 1:**
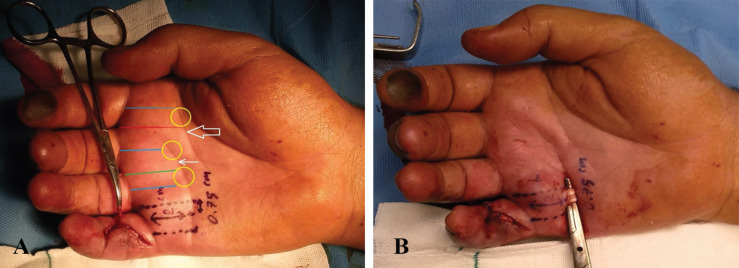
**A: **A 25-year-old man suffers from cuts of the right small finger flexor tendons with glass; Schematic marking of palmar surface markers: Blue lines: The mid-axial lines, red line: the ulnar border distance of Index finger, green line: the radial border distance of ring finger, yellow circles: the exact location of proximal end of the flexor tendons, hallow arrow: the mid-palmar crease, linear arrow: the distal palmar crease and black dots lines: the mid-axial and radial distances of little finger. **B:** The same patient after finding the flexor tendon

**Fig. 2:**
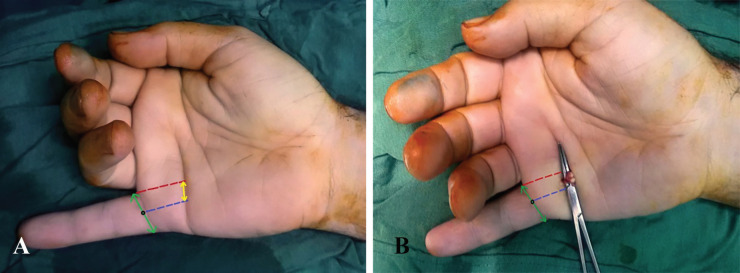
**A:** The 22-year-old man suffered the deep flexor tendon rupture after repairing of the finger injury in another center. Blue dash line: The mid-axial lines, red line: the ulnar border distance of little finger, green arrow: the foot print and the yellow arrow: The location of proximal flexor tendons. **B:** The second patient after detection of the deep flexor tendon

**Fig. 3:**
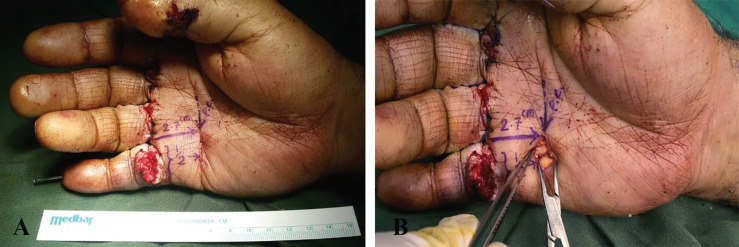
**A:** A 37-year-old man has multiple injuries in Zone 2 due to cuts while working with a saw machine. **B:** After exploration of his deep flexor tendon

To determine the cut tendon end in the palm, the following criteria were used for the little to the index fingers: Two lines were drawn from the radial and finger midaxial edges towards the palm for the little and middle fingers. The interval between the two lines on the palm distal crease would be the location of the proximal flexor tendon end. In the middle finger, the intersection of the finger midaxial line on the distal palmar crease would be the location of the proximal tendon end. In the index finger, the ulnar edge and the finger midaxial line were drawn towards the mid palmar crease, and the interval between these two lines at the intersection with the middle crease will be the proximal tendon end (Figures 1-3).

## DISCUSSION

Zone 2 was introduced as the “no man’s land” for the first time in the Bunnell’s Surgery Textbook, with its anatomical location between the distal palmar crease and the middle finger crease.^8^ Today, this zone includes the area between the A_1_ proximal pulley and the flexor digitorum superficialis (FDS) junction in most definitions. Repairs in Zone 1 are mentioned to be preferable to repairs in Zone 2 in many studies.^1^^,^^2^ Rigo examined 291 patients with flexor tendon laceration and showed that cases with simultaneous damage of FDS and FDP in Zone 2 had less active-rom after 8 weeks.^2^

Repair of the cut FDS in Zone 2 is controversial, although some have better results in tendon gliding and ultimate performance.^9^^,^^10^ Surgery of this zone relies on knowing its fine anatomy, including the location of pulleys. Doyle *et al.* have already identified the position of pulleys A_1_, A_2_, and A_3_, which form palmar plates in distal interphalangeal (DIP), proximal interphalangeal (PIP), metacarpophalangeal (MCP) joints, respectively.^11^^,^^12^ The FDS tendon is branched in pulley A_1_ forming the chiasm of Camper at the depth of the FDP tendon.^13^

The FDS junction is between the first and the mid one-third of the middle phalanx,^14^^,^^15^ while the flexor digitorum profundus (FDP) is at the beginning of the last phalanx.^15^ In Gordon’s study,^7^ the structure of pulleys was determined based on the surface markers of the fingers. In this study, performed on 12 hands-on cadavers, the flexor zones and subzones were precisely determined. Gordon intended to precisely locate the pulley system using the fingers’ surface markers so that hand surgeons can access the injured pulley with limited incisions in the specified areas, but quick and easy remembering of a large amount of palm and hand anatomical details is difficult for surgeons during the operation. In addition, this classification cannot be of much assistance in locating the proximal end of the tendon when the proximal end is pulled back towards the palm.^1^

Nickle studied the junction of FDS tendon to the proximal interphalangeal crease in six pairs of hands-on cadavers.^16^ He reported its location at about 3.22 mm more distal than the proximal interphalangeal crease, but this study only addressed the FDS junction with no description of the Zone 2 injuries and the position of flexor tendons in the palm. The present study examines the position of deep tendons in relation to the surface markers of the palm rather than the previously identified bone markers.^17^

## CONCLUSION

The precise position of flexor tendons can be easily determined in the palm according to palm creases and finger sides. Muscle contraction may retract cut FDP tendons proximally towards the origin of lumbrical muscles in the palm (palmar arc). It is sometimes hard to find the proximal end with the available instruments and to pull it back distally from under the pulleys. On the other hand, damaging the neurovascular bundle and other structures is also a concern. Therefore, this study can help hand surgeons to directly reach the proximal end of the cut tendon through a limited transverse incision in the palm. This guide is very easy to learn, prevents large and extra incisions, and accelerates the surgeons’ performance.

## References

[1] Elhassan B, Moran SL, Bravo C, Amadio P (2006). Factors that influence the outcome of zone I and zone II flexor tendon repairs in children. J Hand Surg Am.

[2] Rigo IZ, Rokkum M (2016). Predictors of outcome after primary flexor tendon repair in zone 1, 2 and 3. J Hand Surg Eur Vol.

[3] Nicholson LT, Hill JR, McKnight B, Heckmann N, Stevanovic M, Ghiassi A (2019). Redefining Zone II: Anatomy of the Flexor Digitorum Superficialis Insertion. Hand (N Y).

[4] Boyes H ( 1964). Joseph Bunnell’s surgery of the hand.

[5] Verdan CE (1960). Primary repair of flexor tendons. JBJS.

[6] Bruner JM (1967). The zig-zag volar-digital incision for flexor-tendon surgery. Plast Reconstr Surg.

[7] Gordon JA, Stone L, Gordon L (2012). Surface markers for locating the pulleys and flexor tendon anatomy in the palm and fingers with reference to minimally invasive incisions. The Journal of Hand Surgery.

[8] Newmeyer WL 3rd, Manske PR (2004). No man’s land revisited: the primary flexor tendon repair controversy. J Hand Surg Am.

[9] Moriya K, Yoshizu T, Maki Y, Tsubokawa N, Narisawa H, Endo N (2015). Clinical outcomes of early active mobilization following flexor tendon repair using the six-strand technique: short- and long-term evaluations. J Hand Surg Eur Vol.

[10] Pike JM, Gelberman RH (2010). Zone II combined flexor digitorum superficialis and flexor digitorum profundus repair distal to the A2 pulley. J Hand Surg Am.

[11] Drake R, Drake RL, Vogl W, Mitchell AW (2004). Gray’s anatomy of the human body.

[12] Doyle JR (1990). Anatomy and function of the palmar aponeurosis pulley. J Hand Surg Am.

[13] Doyle JR (1988). Anatomy of the finger flexor tendon sheath and pulley system. J Hand Surg Am.

[14] Beasley RJ (2003). Beasley’s surgery of the hand.

[15] Moore K, Agur A (2002). Essential clinical anatomy.

[16] Nicholson LT, Hill JR, McKnight B, Heckmann N, Stevanovic M, Ghiassi A (2019). Redefining Zone II: Anatomy of the Flexor Digitorum Superficialis Insertion. Hand (N Y).

[17] Doyle J, Blythe W (1974). Blythe Macroscopic and functional anatomy of the flexor tendon sheath. J Bone Joint Surg.

